# Functional analysis of the intracellular survival of *Mycobacterium avium* subsp. *paratuberculosis* in THP-1 cells using CRISPR interference

**DOI:** 10.1128/jb.00244-25

**Published:** 2025-09-12

**Authors:** Jun Ho Lee, Eun-Seo Lee, Su Min Kyung, Xi-Rui Xiang, Hyun-Eui Park, Min-Kyoung Shin, Han Sang Yoo

**Affiliations:** 1 Department of Infectious Disease, College of Veterinary Medicine, Seoul National University, Seoul, Republic of Korea; 2 BK21 FOUR Future Veterinary Medicine Leading Education and Research Center, College of Veterinary Medicine, Seoul National University, Seoul, Republic of Korea; 3 Research Institute for Veterinary Science, College of Veterinary Medicine, Seoul National University, Seoul, Republic of Korea; 4 Department of Microbiology, College of Medicine, Yonsei University Wonju, Wonju, Republic of Korea; 5 Department of Microbiology and Convergence Medical Science, College of Medicine, Gyeongsang National University, Jinju, Republic of Korea; University of Illinois Chicago, Chicago, Illinois, USA

**Keywords:** *Mycobacterium avium *subsp. *paratuberculosis*, CRISPR interference, MAP mutant, THP-1 macrophage, intracellular survival

## Abstract

*Mycobacterium avium* subsp. *paratuberculosis* (MAP) is a causative agent of Johne’s disease in ruminants and a potential zoonotic agent linked with Crohn’s disease in humans. Despite the possible risk to public health, few studies have focused on the virulence of MAP against human macrophages. Therefore, a functional analysis of mycobacterial genes associated with virulence, especially the intracellular survival of MAP, was performed after infection of MAP CRISPR interference (CRISPRi) mutants in the human THP-1 macrophages. MAP mutants were targeted to four genes (*mdh*, *pknG*, *MAP1981c*, and *icl*). The optimal concentration of anhydrotetracycline (ATc) was determined to be 5 µg/mL by measuring the survival of the cells and the downregulation of gene expression levels in the cells up to Day 3. The clump formation and intracellular survival of MAP were investigated using transmission electron microscopy and the colony-forming units, respectively. The clump formation of MAP mutants induced by CRISPRi was decreased in THP-1 macrophages at 24 and 72 h post-infection. The survival rates of the MAP mutants significantly decreased with increasing ATc concentration and time course of infection in MAP*-mdh*KD, *MAP1981c*KD, and MAP*-icl*KD. Conversely, the survival rate of THP-1 macrophages increased with increasing ATc concentration. Our results suggest that these genes might be closely related to MAP virulence along with intracellular survival in THP-1 macrophages. These data can provide novel insights into the utilization of CRISPRi in further research on MAP virulence by exploring intracellular survival using mycobacterial genes related to the virulence of MAP during host infection.

**IMPORTANCE:**

Johne’s disease, caused by *Mycobacterium avium* subsp. *paratuberculosis* (MAP) is a worldwide issue in the dairy industry and has a possible connection to Crohn’s disease (CD) in humans. Despite its potential contribution to the etiology of CD, there have been few studies focusing on the virulence of MAP against human macrophages. In the current study, we investigated MAP virulence along with intracellular survival in human THP-1 macrophages using functional analysis of MAP CRISPR interference (CRISPRi) mutants at the knockdown of genes associated with mycobacterial virulence. The identified potential genes represent novel candidate classes that could be necessary for MAP virulence by exploring intracellular survival during host infection and could provide novel insights for future studies on the utilization of CRISPRi.

## INTRODUCTION

*Mycobacterium avium* subsp. *paratuberculosis* (MAP), the primary causative agent of Johne’s disease (JD), continues to pose a significant threat to the livestock industry by inducing chronic enteritis in ruminants (1, 2). Beyond its veterinary significance, MAP also holds public health implications (3), as it has been suggested to be a potential etiological agent of Crohn’s disease (CD) in humans (4–7). Central to the pathogenesis of MAP is its ability to persist within host macrophages, facilitating immune evasion and chronic infection (8–11). This intracellular persistence, alongside the capability to evade and modulate host immune defenses, has driven considerable research on the mechanisms underlying MAP’s survival within host cells (10, 12–14). Consequently, understanding these processes and identifying pivotal genes involved in MAP’s intracellular survival are crucial for developing new vaccines and therapeutic interventions.

Previous studies have implicated several MAP genes in virulence and intracellular persistence based on indirect or preclinical evidence (15–19). Among these, we selected four genes (*mdh*, *pknG*, *MAP1981c*, and *icl*) based on their putative involvement in facilitating MAP’s survival within host cells, modulating immune responses, and sustaining infection. First, *mdh* (malate dehydrogenase) is known to play a pivotal role not only in carbohydrate metabolism through the TCA cycle but also in triggering immune responses within the host (20, 21). Of particular note, the detection of *mdh*-specific antibodies in the serum of CD patients suggests that this gene may be involved in MAP’s ability to modulate or disrupt host immune pathways (22). Second, *pknG* (protein kinase G) has been well characterized in other mycobacteria, where it inhibits phagosome-lysosome fusion in macrophages, thereby promoting intracellular persistence and subverting host immune clearance mechanisms (23, 24). This raises the possibility that *pknG* also confers a similarly vital role in evading the host’s immune mechanisms in MAP. Third, *MAP1981c* is suggested to act as a nucleic acid-binding protein, thereby possibly participating in DNA synthesis or replication, even though its precise function remains unknown (25). It is known to be a highly immunogenic antigen in both cattle with JD and patients with CD, implying a potential role in not only virulence but also in the modulation of host immune responses (25). Notably, it exhibited strong immunoreactivity with IgG and IgM in sera from CD patients (25). Furthermore, MAP1981c has been shown to stimulate robust immune activation in Peyer’s patches, the primary site of MAP entry in the intestine, and is reactive to sera from MAP-infected calves (26). These findings imply a potential role in initiating or modulating host immune responses during early infection stages. Finally, *icl* (isocitrate lyase), a key enzyme in the glyoxylate pathway, is believed to be vital for overcoming nutrient limitations inside host cells (27–30). This pathway is essential for intracellular pathogens that encounter nutrient restriction within macrophages (28, 31). In MAP, *icl* is believed to support long-term persistence by maintaining metabolic flexibility during chronic infection (30). Despite mounting evidence for these genes’ roles in virulence such as encompassing intracellular survival, immune modulation, and metabolic adaptation—direct and comprehensive studies confirming their precise contributions to long-term survival and sustained infection in host cells remain limited.

Recent advances in CRISPR interference (CRISPRi) technology, which allows for targeted repression of gene expression (transcriptional silencing) in bacteria, have provided a valuable tool for the direct functional analysis of specific or target genes (32, 33). In contrast to gene deletion (knockout) strategies, CRISPRi offers reversible and precise control over gene expression—particularly advantageous when examining essential genes linked to bacterial viability (32, 34, 35). Initially applied in *Mycobacterium tuberculosis* (Mtb) (36), CRISPRi has enabled targeted silencing of multiple genes in mycobacteria, thereby accelerating functional genomics studies (37–39). However, although this technology has been adapted for various mycobacterial species, its application to MAP remains largely unexplored, and there are no reports on its use to assess MAP’s virulence within host cells.

Here, we employed a CRISPRi approach in MAP to generate and analyze mutant strains of the four aforementioned genes, focusing on their intracellular survival and localization within THP-1 macrophages following targeted gene silencing. Our findings underscore the potential of CRISPRi as a novel experimental platform for clarifying MAP’s pathogenic mechanisms and highlight possible gene targets for future vaccine and therapeutic development.

## RESULTS

### Establishment and validation of CRISPRi gene knockdowns in MAP

**Fig 1:**
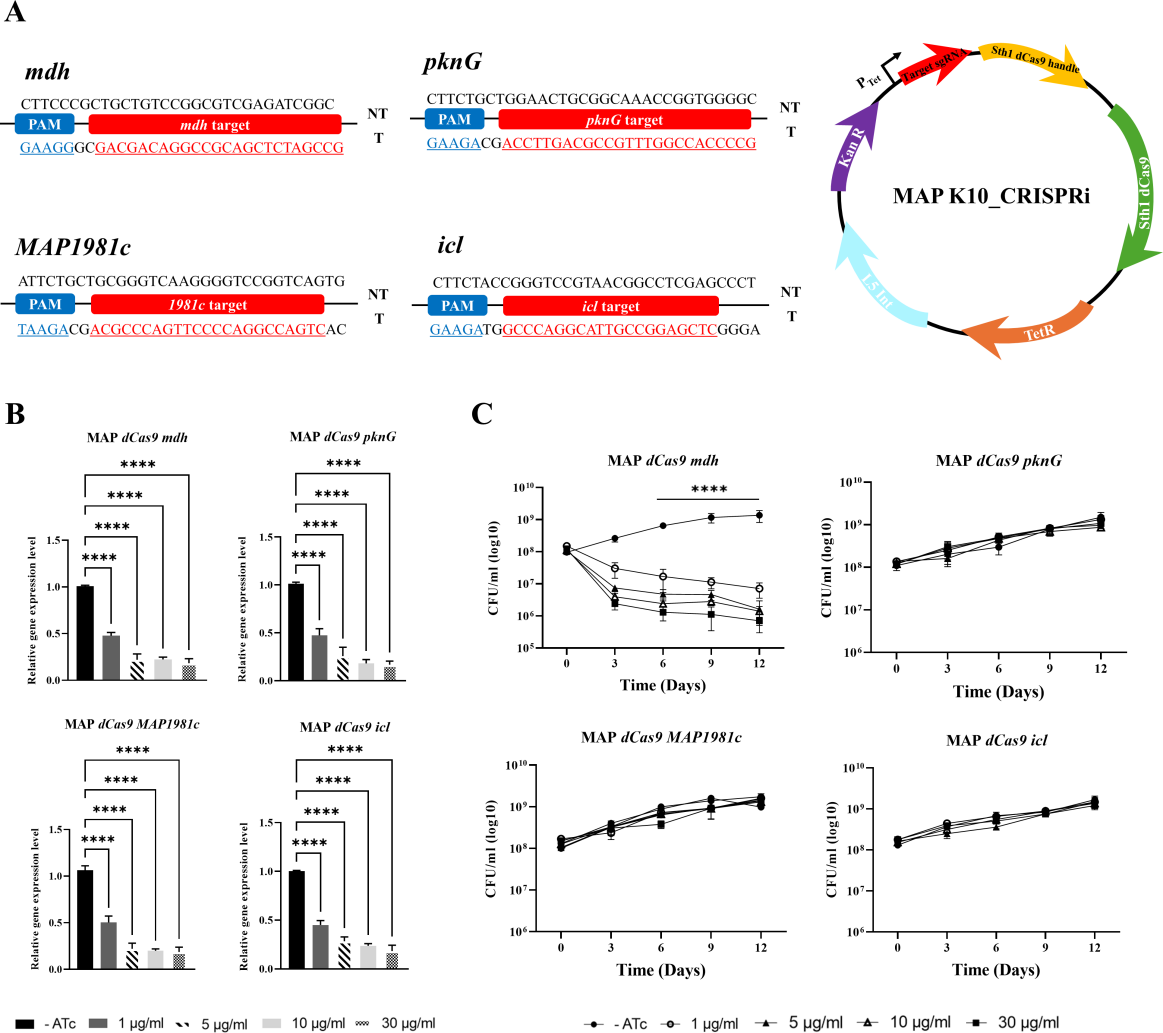
MAP CRISPRi mutant construction and knockdown response. (**A**) CRISPRi target genes and CRISPRi plasmid maps for MAP. sgRNA targets of four MAP virulence-related genes are highlighted in red. Red underlined shows designed sgRNA targeting template strand. The translational start codon, PAM (blue), is also depicted. MAP K10 CRISPRi plasmid expresses target sgRNA (red) and Sth1 dCas9 handle (yellow). Transformants harboring the plasmid were selected based on kanamycin resistance conferred by the KanR (purple). TetR-regulated promoters are transcriptionally activated in the presence of ATc, CRISPRi inducer. The sgRNA target sequences were added to the pLJR965 plasmids for the entire plasmid: a reverse primer and forward primer with an ~25 bp target sequence was added to the 5′ end (red). Analysis of the mRNA transcript levels of four MAP mutant target genes as indicated in Figure (**B**). mRNA levels of each gene after induction with -ATc (control), 1, 5, 10, or 30 µg/mL ATc induction in CRISPRi gene knockdowns on Day 3. (**C**) CRISPRi targeting of four MAP mutant target genes results in a viability determined via CFU counts. MAP mutants were subsequently grown in kanamycin-7H9 broth until they reached the log phase and ATc was supplemented with -ATc (control), 1, 5, 10, or 30 µg/mL. CFUs were observed on kanamycin-7H10 agar. CFU/mL was calculated from colony counts of the control and four MAP mutant target gene knockdown constructs. The fold change values are relative to those of the control uninduced (no ATc), and the data presented as the means ± SDs of at least three biological replicates. Statistical significance was calculated by one-way (**B**) and two-way ANOVA (**C**) with Tukey’s test for multiple comparisons (*P*-value, *<0.05; **<0.01; ***<0.0005; ****<0.0001).

A mycobacterial single-plasmid dCas9Sth1 CRISPRi system was employed to downregulate the expression of selected *M. avium* subsp. *paratuberculosis* (MAP) virulence-related genes, using the pLJR965 plasmid to enhance silencing efficiency (34). Four genes—*mdh*, *pknG*, *MAP1981c*, and *icl*—were targeted with specific primers for MAP mutant construction (Fig. 1A). CRISPRi knockdown constructs were generated following a previously described protocol (30), as illustrated in Fig. 1A. To evaluate the efficacy of these CRISPRi constructs, different concentrations of anhydrotetracycline (ATc; 1, 5, 10, and 30 µg/mL) were examined. On Day 3 post-induction, quantitative real-time PCR (qRT-PCR) showed an approximately twofold and fourfold decrease in gene expression at 1 and 5 µg/mL ATc, respectively, increasing to sixfold or more at 30 µg/mL (Fig. 1B). Subsequently, the impact of these ATc concentrations on bacterial viability was assessed over 12 days in log-phase cultures. Among the four mutants, in the case of MAP*-mdh*KD, approximately 1 log_10_ reduction difference in CFU was observed over 12 days at a concentration of 1 µg/mL, and as the concentration of ATc increased (5–30 µg/mL), the difference in CFU also increased, showing up to more than 2 log_10_ reduction (Fig. 1C). These results indicate that the difference in viability due to gene knockdown became more pronounced when ATc was administered at concentrations of 5–30 µg/mL compared with 1 µg/mL. In contrast, no notable changes in viability were observed in the other three mutants (MAP*-pknG*KD, *MAP1981c*KD, and MAP*-icl*KD) (Fig. 1C).

### Optimization of ATc concentration for THP-1 macrophage infection with MAP CRISPRi mutants

**Fig 2:**
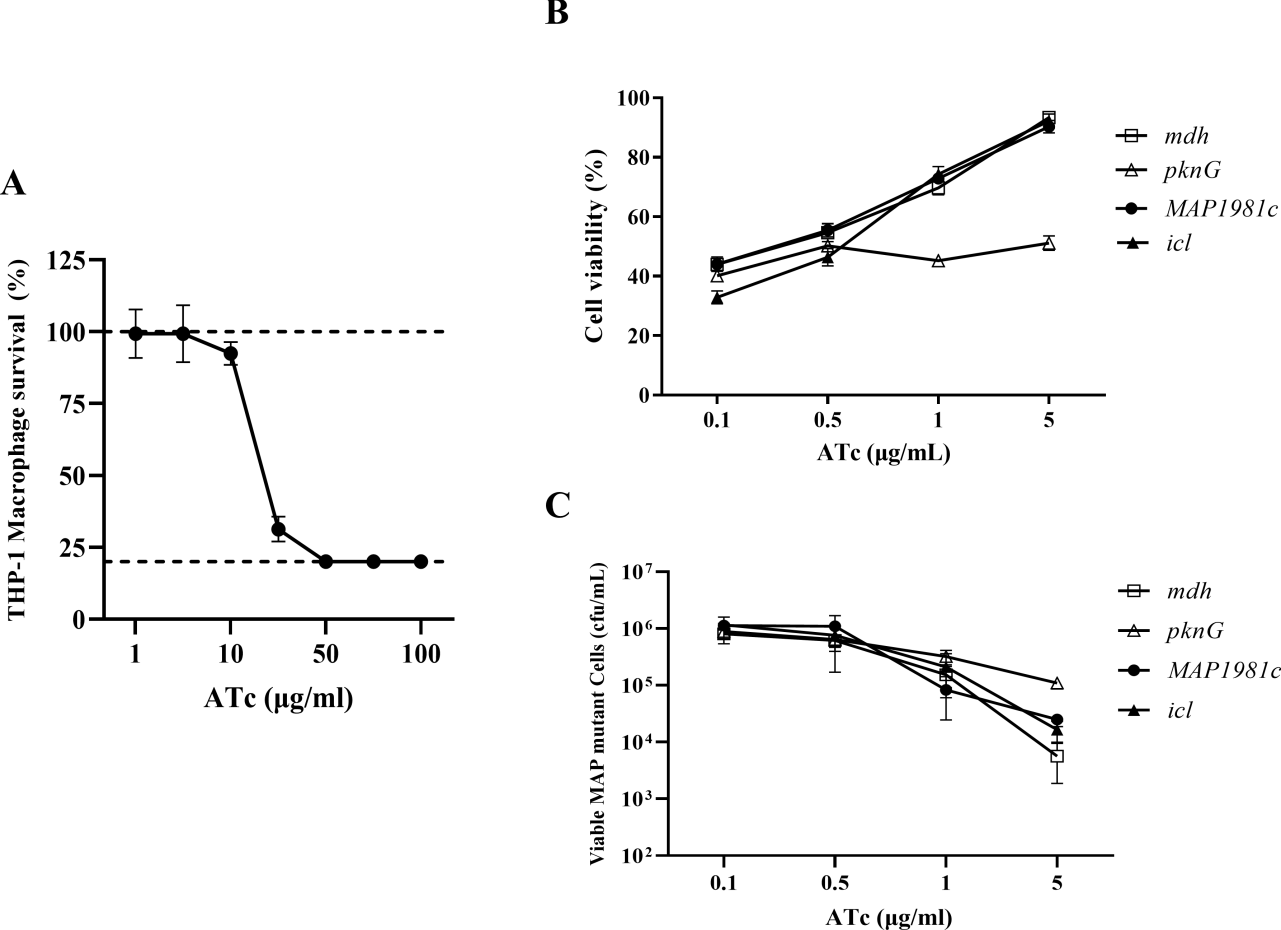
Optimization of ATc concentration for THP-1 macrophages viability and survival rates of MAP CRISPRi mutants. (**A**) The survival of THP-1 treated with increasing concentrations of ATc was determined using WST-8 assay. (**B**) Survival of THP-1 macrophages infected with MAP-expressing four genes-targeting sgRNA for 3 days following the addition of the indicated concentrations of ATc. The survival of THP-1 macrophages was determined using WST-8 assay. The results are presented as percentages relative to the control (no ATc-treated). (**C**) Survival of intracellular MAP-expressing four genes-targeting sgRNA within THP-1 macrophages depending on ATc use after 3 days at the stated ATc concentrations, as detected by CFU counts. CFU spreading was carried out on kanamycin-7H10 agar for each concentration shown in the graph. The results are presented as the means ± SDs of three replicates.

To determine an appropriate concentration of ATc for inducing CRISPRi-mediated gene knockdown in MAP without negatively affecting host cell viability, we first evaluated the cytotoxic effects of increasing ATc concentrations on THP-1 macrophages using the WST-8 assay (Fig. 2A). First, to measure changes in THP-1 macrophage viability using the WST-8 assay, differentiated THP-1 macrophages were cultured in RPMI 1640 medium with an ATc concentration range set from 1–100 µg/mL. At concentrations of 5 µg/mL or lower, no significant changes in cell viability were observed; however, at higher concentrations of 30 µg/mL or more, viability sharply decreased by more than half (Fig. 2A). Next, to assess the effect of ATc on infected macrophages, THP-1 macrophages were infected with MAP CRISPRi mutants targeting four genes (*mdh*, *pknG*, *MAP1981c*, and *icl*), followed by administration of ATc at concentrations ranging from 0.1 to 5 µg/mL. The viability of the infected macrophages using the WST-8 assay was then measured. Three days post-infection, ATc at concentrations up to 5 µg/mL did not induce significant cytotoxicity in the infected macrophages (Fig. 2B). In addition, infection with the MAP*-pknG*KD resulted in relatively significant changes in macrophage viability across different ATc concentrations, whereas infection with the MAP*-mdh*KD, *MAP1981c*KD, and MAP*-icl*KD led to a concentration-dependent marked increase in macrophage viability (Fig. 2B). Notably, at 5 µg/mL, cell viability increased by more than twofold compared with 0.1 µg/mL (Fig. 2B). This suggests a reduction in intracellular bacterial burden. Indeed, when bacterial viability was assessed under the same conditions using CFU counting, the MAP*-mdh*KD, *MAP1981c*KD, and MAP*-icl*KD showed a decreased intracellular bacterial survival (Fig. 2C). In contrast, MAP-*pknG*KD exhibited a relatively modest reduction in intracellular bacterial survival at 5 µg/mL, indicating a less pronounced impact on bacterial viability compared with the other knockdown strains (Fig. 2C).

### Temporal analysis of intracellular survival in MAP CRISPRi mutants

**Fig 3:**
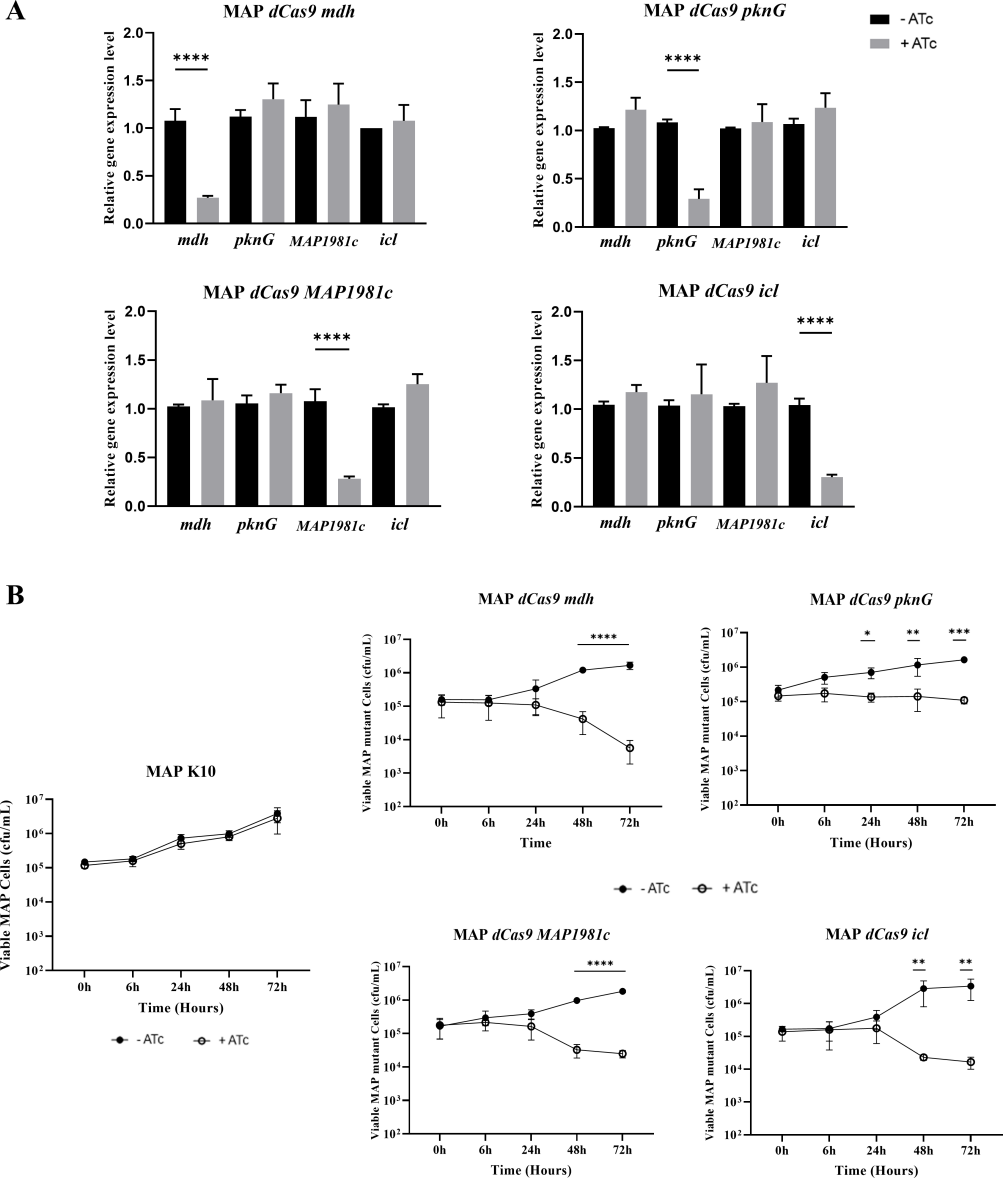
CRISPRi-mediated gene silencing and survival rates of MAP mutants in THP-1 macrophages. (**A**) qRT-PCR analysis of gene expression profiles in THP-1 macrophages infected with MAP CRISPRi mutants targeting *mdh*, *pknG*, *MAP1981c*, or *icl*. For each mutant strain, expression levels of the targeted gene and the remaining three non-targeted genes were assessed on Day 3 post-infection under ATc-induced (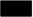, +ATc, 5  µg/mL) and uninduced (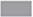,-ATc) conditions. Fold change values are shown relative to the uninduced control group. (**B**) Survival rates of MAP CRISPRi mutants and MAP K10 in THP-1 macrophages throughout the time course of infection. Survival of intracellular MAP-expressing *mdh*, *pknG*, *MAP1981c*, and *icl* gene-targeting sgRNA and MAP K10 within THP-1 macrophages over time with 5 µg/mL ATc, as detected by CFU counts. CFU spreading was performed on kanamycin-7H10 agar for each concentration shown in the graph. The black and white circles denote supplementation with and without ATc, respectively. The data are presented as the means ± SDs of at least three biological replicates. Statistical significance was calculated by (**A**) one-way and (**B**) two-way ANOVA with Tukey’s test for multiple comparisons (*P*-value, *<0.05; **<0.01; ***<0.0005; ****<0.0001).

In MAP-infected THP-1 macrophages, the expression of virulence-associated genes (*mdh*, *pknG*, *MAP1981c*, and *icl*) was suppressed using CRISPRi, and the impact of these genes on intracellular survival was evaluated over time. First, the optimal ATc concentration (5 µg/mL), established on prior experiments, was administered on Day 3 post-infection, and the expression levels of each target gene were assessed by qRT-PCR (Table S1). As a result, all four MAP target genes showed more than a threefold reduction in expression compared with the untreated control group, and the downregulation of each target gene did not affect the expression of the remaining three target genes (Fig. 3A).

Subsequently, under the same gene-silencing conditions, the survival of MAP mutants within THP-1 cells was monitored over a 72-h period by counting CFUs. During the first 24 h of infection, no significant differences were observed between the four mutant strains and the control. However, after 24 h, the CFU counts of the MAP*-mdh*KD, *MAP1981c*KD, and MAP*-icl*KD decreased sharply by more than 1 log_10_, suggesting that these genes play critical roles in intracellular survival. In contrast, MAP*-pknG*KD showed a gradual decrease in CFU counts after 24 h; however, the extent of the reduction in intracellular survival was relatively modest compared to the other three mutants (Fig. 3B). These results suggest that MAP*-mdh*KD, *MAP1981c*KD, and MAP*-icl*KD play more critical roles during the intracellular infection process of MAP, whereas MAP*-pknG*KD also contributes to intracellular survival, albeit to a lesser extent.

### Intracellular survival from mycobacterial clump formation in THP-1 macrophages

**Fig 4:**
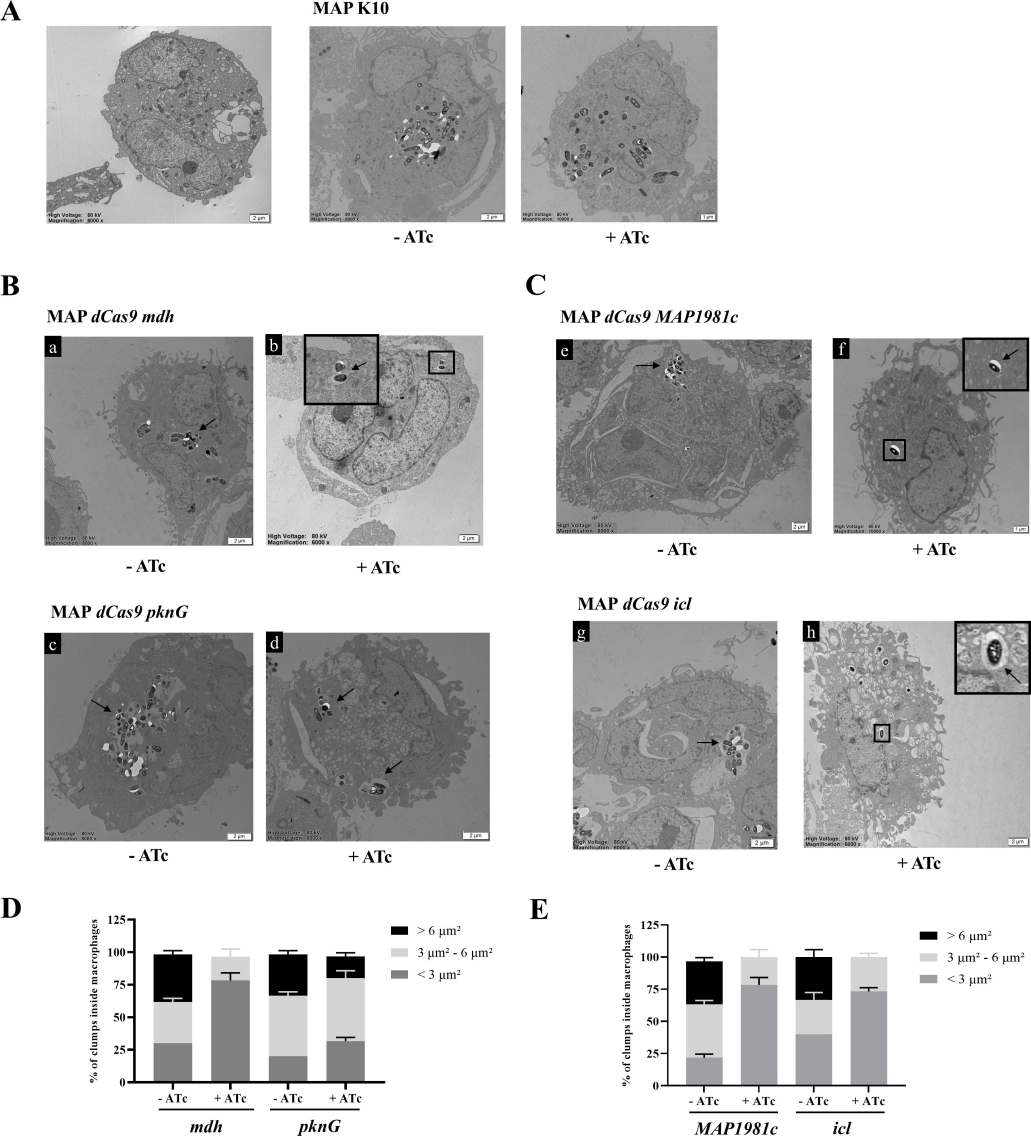
Localization of four MAP CRISPRi mutants at 24 h.p.i. using transmission electron microscopy (TEM). (**A**) Representative TEM images of uninfected macrophages and THP-1 macrophages infected with MAP K10, depending on the ATc use. Scale bar, 1 or 2 µm. (**B**) 6,000- or 8,000-fold TEM magnification of images of THP-1 macrophages infected with MAP*-mdh*KD or MAP*-pknG*KD, depending on ATc use. An intracellular clump of MAP*-mdh*KD and MAP*-pknG*KD inside a large phagocytic vesicle and the membrane of the phagocytic vesicle is indicated with a black arrow in a-d MAP*-mdh*KD and MAP*-pknG*KD. Scale bar, 2 µm. (**C**) 6,000- to 10,000-fold TEM magnification of images of THP-1 macrophages infected with *MAP1981c*KD and MAP*-icl*KD, depending on ATc use. An intracellular clump of a *MAP1981c*KD and MAP*-icl*KD inside a large phagocytic vesicle and the membrane of the phagocytic vesicle is indicated with a black arrow in a, b *MAP1981c*KD and MAP*-icl*KD. Scale bar, 1 or 2 µm. (**D, E**) Results of the analysis of the percentages of clumps of different sizes inside the macrophages at 24 h p.i. in MAP*-mdh*KD, MAP*-pknG*KD, *MAP1981c*KD, and MAP*-icl*KD, depending on ATc use. The results represent the means ± SDs of triplicate preparations.

**Fig 5:**
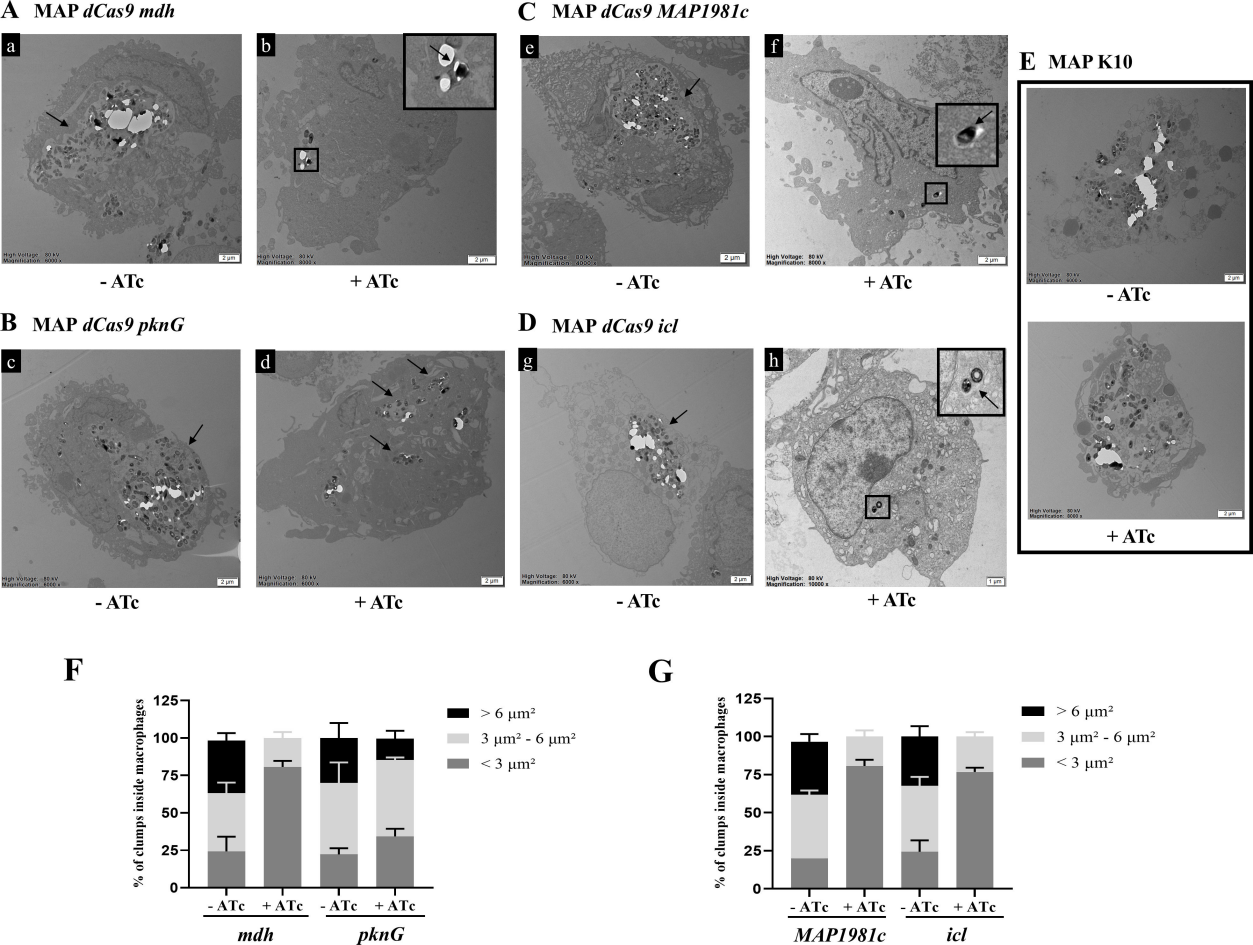
Localization of four MAP mutants and MAP K10 at 72 h.p.i. using transmission electron microscopy (TEM). (**A–D**) 4,000- to 10,000-fold TEM magnification images of THP-1 macrophages infected with MAP mutants at 48 h.p.i., depending on ATc use. The membrane of the phagocytic vesicle is indicated with a black arrow. An intracellular clump of a, c, e, g MAP mutants and individual bacilli of b, f, h MAP mutants inside a phagocytic vesicle. Scale bar, 1 or 2 µm. (**E**) Representative TEM images of THP-1 macrophages infected with MAP K10, depending on the ATc use. Scale bar, 2 µm. (**F, G**) Results of the analysis of the percentages of clumps of different sizes inside the macrophages at 72 h.p.i. in MAP*-mdh*KD, MAP*-pknG*KD, *MAP1981c*KD, and MAP*-icl*KD, depending on ATc use. The results represent the means ± SDs of triplicate preparations.

To further investigate the phenotypic consequences of CRISPRi-mediated gene silencing, the intracellular localization and aggregation patterns of MAP CRISPRi mutants were evaluated using transmission electron microscopy (TEM) at 24 and 72 h post-infection (h.p.i.) (Fig. 4 and 5). The TEM images clearly revealed fewer clumps of individual bacilli inside the phagocytic vesicles of THP-1 macrophages infected with MAP*-mdh*KD, *MAP1981c*KD, and MAP*-icl*KD, depending on the use of ATc, at 24 h.p.i. (Fig. 4B and C). In contrast, TEM showed that MAP*-pknG*KD produced small clump formation inside the phagocytic vesicles of THP-1 macrophages when gene expression was downregulated (Fig. 4B). At 24 h.p.i., between 30% and 40% of the bacilli observed inside THP-1 macrophages infected with all MAP mutants without ATc were organized in clumps bigger than 6 µm² (Fig. 4D and E). At 72 h.p.i., similar to 24 h.p.i., the presence of fewer clumps of individual bacilli in phagocytic vesicles of THP-1 macrophages was confirmed through gene expression downregulation of MAP*-mdh*KD, *MAP1981c*KD, and MAP*-icl*KD (Fig. 5A,C and D). Moreover, similar to the observations at 24 h.p.i., approximately 30%–40% of intracellular bacilli in THP-1 macrophages infected with each of the MAP mutants were found to form clumps exceeding 6 µm² in size (Fig. 5F and G). However, in the case of MAP*-pknG*KD, despite gene expression downregulation, small clumps from individual bacilli formed at 72 h.p.i. (Fig. 5B). In all mutants without ATc treatment, large clumps remained prominent at both time points. Similarly, MAP K10 formed intracellular clumps regardless of ATc treatment or infection time (Fig. 4A and 5E). These results indicate that MAP*-mdh*KD, *MAP1981c*KD, and MAP*-icl*KD may facilitate MAP aggregation and survival within macrophages, whereas MAP*-pknG*KD appears to play a lesser role in clump formation.

## DISCUSSION

In this study, using the pLJR965-based CRISPRi system, we successfully established a gene downregulation platform for MAP, which remained functional during intracellular infection of THP-1 macrophages, enabling investigation of gene-specific contributions in bacterial survival and pathogenicity. MAP is a slow-growing intracellular pathogen in ruminants with growing attention to link with CD in humans (40–42) and presents significant technical challenges for genetic manipulation (2, 43). While CRISPRi technology has been applied in other mycobacterial species (37, 44, 45), its implementation in MAP has not yet been validated. Here, we developed a dCas9-based CRISPRi platform using the pLJR965 vector and demonstrated its successful application both *in vitro* and within human THP-1 macrophages. Our system enabled inducible repression of target genes during infection and allowed us to evaluate their roles in bacterial persistence and intracellular behavior.

A dCas9-based CRISPRi system was applied to MAP to precisely downregulate the expression of specific genes and assess changes in bacterial survival within a human-derived THP-1 macrophage infection model. Although the pLJR965-based CRISPRi platform has been optimized in previous studies for Mtb (36), its direct application to MAP has been extremely limited. Therefore, to successfully implement the CRISPRi system in MAP, we used the pLJR965-based CRISPRi platform in which ATc serves as an inducer to guide the sgRNA-dCas9 complex to the target gene, thereby interfering with RNA polymerase progression and downregulating gene expression. Using the generated MAP CRISPRi mutant, we first aimed to identify the optimal balance between gene expression downregulation level and THP-1 cytotoxicity. As a result, we found that an ATc concentration of 5 µg/mL effectively downregulated gene expression level in the MAP mutant without significantly reducing host cell viability. Compared with previous studies conducted on mycobacteria (38, 46, 47), this study exhibited differences in terms of the growth rate of the pathogen, target cell for infection, and the process of ATc optimization. In prior studies involving *M. abscessus* (Mab), optimization was conducted by administering a range of ATc concentrations (0–500 ng/mL), where growth pattern assessments revealed that concentrations of ATc ≥150 ng/mL inhibited bacterial growth (38). Additionally, previous research on Mtb employed a CRISPRi screening approach in an infection model for the first time, similar to this study, but used beta score calculations to evaluate CRISPRi induction and compared axenic culture with a murine bone marrow-derived macrophage model to assess the essentiality of Mtb-specific genes for intracellular survival (46). In contrast, previous CRISPRi work in *M. smegmatis* utilized the pLJR962 plasmid for construct development and assessed the intracellular survival of multiple sgRNA candidates of mutants via CFU enumeration within J774 murine macrophages (47). However, ATc concentration determined in this study is comparable to the condition reported in Mtb models (44) and holds significance as it represents stable application and establishment of the CRISPRi system, and optimization tailored to the characteristics of MAP virulence and the THP-1 cell condition.

Under the established CRISPRi conditions (5  µg/mL of ATc), we successfully targeted four genes—*mdh*, *pknG*, *MAP1981c*, and *icl*—each implicated in MAP pathogenicity and intracellular survival. Notably, downregulation of each target gene did not alter the expression levels of the remaining targets, confirming the specificity of CRISPRi-mediated silencing and minimizing concerns about potential off-target effects. In addition, this study employed a combination of TEM imaging and CFU counts to quantitatively assess the presence and size of clumps formed within macrophages, thereby enabling a multifaceted evaluation of MAP survival mechanisms. The presence, size, and morphology of intracellular clumps observed via TEM serve as critical indicators of mycobacteria’s ability to survive and persist within host macrophages (9, 48). Previous studies on Mab have demonstrated that macrophages can phagocytose large bacterial clumps consisting of five or more bacilli within a single phagosome, and that these clumps are closely associated with macrophage death, enhanced release of proinflammatory cytokines, and increased bacterial virulence (48). These findings suggest that the physical form of mycobacteria—specifically the formation of intracellular clumps—can serve as a proxy for evaluating bacterial pathogenicity and host defense evasion capacity. Based on this criterion, we adopted a similar approach to assess whether the downregulation of MAP virulence-related genes affects the ability of bacilli to form clumps within macrophages. By quantitatively analyzing the morphology of clumps in TEM images and the number of CFU, we aimed to determine the functional relevance of each gene in mediating MAP’s intracellular aggregation and persistence, thereby providing new insights into the mechanistic links between clump formation and pathogenic survival strategies.

Using TEM, we observed the formation of clumps of four MAP mutants at 24 and 72 h post-infection (h.p.i.) in the control group, suggesting that these clumps resulted from the engulfment of bacilli and replication inside the phagocytic vesicles through intracellular survival. In contrast, we observed the presence of individual bacilli of three MAP mutants (MAP*-mdh*KD, *MAP1981c*KD, and MAP*-icl*KD) at 24 and 72 h.p.i. in the treatment groups, suggesting that the percentage of clump formation inside the phagocytic vesicles decreased when each gene was downregulated. As *mdh* is essential for the TCA cycle and mycobacterial growth under hypoxic conditions (20, 49), its downregulation likely interfered with the ability to replicate efficiently within the host cell, thereby reducing the formation of intracellular clumps. Similarly, *MAP1981c*, identified as a novel immunocompetent antigen, plays a critical role in immune modulation during the early stages of infection (25). The downregulation of this gene likely disrupted the ability to activate immune responses and survive within the macrophage in early infection, leading to a decrease in clump formation. Finally, *icl*, which is key to fatty acid metabolism in the glyoxylate cycle (28, 50), has been shown to support mycobacterial persistence in chronic infection (30). Its downregulation likely impaired the mycobacterial metabolic adaptation to the host environment, reducing its ability to form clumps and persist inside macrophages.

Time course infection experiments demonstrated that three MAP mutants (MAP*-mdh*KD, *MAP1981c*KD, and MAP*-icl*KD) caused no significant difference in viable intracellular bacteria within 24 h but resulted in an approximately 2 log_10_ reduction by 48 and 72 h, respectively. Compared with the initial infection, MAP*-pknG*KD presented a constant survival rate for up to 24 h, after which it slightly decreased but no more than a 1 log_10_ reduction. Three target genes that led to a decrease in the survival rate might be closely related to the virulence of MAP in THP-1 macrophages. These results are in contrast to previous studies in which a *pknG* deletion mutant showed a significant reduction in survival rate on Day 6 infection with bovine monocyte-derived macrophages (MDMs) (51). Another research demonstrated that the viability of *icl* gene knockout mutant significantly decreased in bovine MDMs, supporting a potential link between our findings and previous studies (30).

Taken together, this study clearly demonstrates, through TEM and CFU analyses, that the downregulation of *mdh*, *MAP1981c*, and *icl* significantly impairs intracellular clump formation, which leads to reduced mycobacterial viability. These findings reaffirm that clump formation is a critical phenotype for intracellular survival of MAP and newly highlight that *MAP1981c*, which is prominently expressed during early infection (25), along with *mdh* and *icl*, which are involved in stress adaptation (20, 52), are directly linked to clump formation. However, the relatively modest reduction in MAP*-pknG*KD survival suggests that *pknG* may play a less dominant role in early intracellular survival in THP-1 macrophages, or its contribution might be environmental condition-dependent, varying across different host cell lines or infection stages. Further investigation will be required to elucidate the precise role of *pknG* under varying host-pathogen interaction conditions.

This study holds significant value in that it empirically demonstrates the correlation between the intracellular functions of virulence-associated genes and clump formation in a THP-1 macrophage infection model using the CRISPRi system in MAP. However, since the THP-1 cell line does not fully represent the physiological environment of primary human macrophages, when interpreting certain differences, such as those observed in the MAP*-pknG*KD results, should be interpreted with consideration of the host cell origin and immunological background. To strengthen the relevance of these findings, future studies using bovine macrophages, the primary host cells of MAP in natural infections, will be essential for validating and extending the insights gained from the THP-1 model. While the CRISPRi system employed in this study allowed us to investigate gene-specific functions in MAP during macrophage infection, its application in *in vivo* settings remains technically challenging. This is primarily due to the requirement for continuous expression of the dCas9-sgRNA system, often dependent on ATc induction, which may be difficult to maintain or regulate in animal models (53). Nevertheless, similar to Mtb, the effective intracellular penetration of ATc holds potential for enabling CRISPRi-mediated transcriptional control of MAP *in vivo*, including in animal infection models (54). In particular, the phenotypic changes observed on the host side, such as clump formation suppression and increased macrophage viability, highlight the potential of this platform not only for bacterial functional genomics but also for interrogating host responses, which could be further extended to primary macrophages or *in vivo* models in future investigations. However, further development of inducible or transient systems suitable for animal models is believed to be necessary to apply these research results to *in vivo* settings. In addition, although knockout (KO) mutants have been developed and used to identify essential gene functions in mycobacterial species (30, 55, 56), the CRISPRi system offers a key advantage over KO methods by enabling reversible and tunable control of gene expression (32, 34). Specifically, considering that gene deletion in KO mutants may lead to compensatory mechanisms or secondary mutations during long-term culture (57, 58), the use of an inducible system such as CRISPRi allows for useful control of gene expression (32). This enables functional analysis of target genes while maintaining bacterial physiology, offering a more precise means to investigate clump formation and intracellular survival mechanisms through finely tuned regulation of gene expression. In conclusion, this study enhances the understanding of MAP pathogenicity and the potential for therapeutic target discovery by optimizing the application of the CRISPRi system in MAP and analyzing the functional roles of virulence-associated genes from the perspective of clump formation.

## MATERIALS AND METHODS

### Bacterial strains and culture conditions

The wild-type MAP strain used in this study was reference strain MAP ATCC BAA-968 (K10, isolated from cattle feces), and routinely cultured in Middlebrook 7H9 broth supplemented with oleic-albumin-dextrose-catalase (OADC) and Mycobactin J. Four types of MAP CRISPRi mutants (MAP*-mdh*KD, MAP*-pknG*KD, *MAP1981c*KD, and MAP*-icl*KD) and K10 were cultured at 37°C in Middlebrook 7H9 broth (Beckton Dickinson Biosciences, Sparks, MD, USA) supplemented with 0.2% glycerol, 0.05% Tween 80, Mycobactin J (2 mg/L, Allied Monitor, Fayette, MO), and 10% OADC enrichment (Beckton Dickinson Biosciences). Middlebrook 7H10 agar supplemented with 0.5% glycerol, Mycobactin J (2 mg/L), and 10% OADC enrichment (Beckton Dickinson Biosciences) was used. When necessary, the medium was supplemented with 50 µg/mL kanamycin and/or 5 µg/mL anhydrotetracycline (ATc) (Sigma‒Aldrich, Co., St. Louis, MO, USA) dissolved in 70% ethanol and added to the experiments at the stated concentrations.

### Construction of MAP CRISPRi knockdown mutants

CRISPRi knockdown mutants were constructed targeting the non-template strand of four genes (MAP2541c: *mdh*, MAP3893c: *pknG*, *MAP1981c*: hypothetical protein, MAP3961: *icl*) using the CRISPRi backbone plasmid, pLJR965 (Addgene plasmid #11563, http://n2t.net/addgene:115163) (36). To better compare the downregulation of the 4 target genes, PAM motifs with a predicted strength of 145- or 216-fold luciferase repression were selected (36). In brief, based on the sgRNA design process, the PAM sequence was identified within the target gene, after which the sgRNA targeting sequence was extracted; this sequence was approximately 20–25 nucleotides adjacent to the PAM sequence. To clone the sgRNA targeting sequences, complementary sgRNA targeting oligos (N20-25) were annealed at 95°C for 5 min using each sgRNA promoter and dCas9 handle sequence. The resulting product was ligated into the gel-purified BsmBI-digested CRISPRi vector backbone. These constructs were subsequently transformed into *E. coli* (DH5α) competent cells, which were subsequently cultured in Luria-Bertani (LB) broth supplemented with kanamycin during the early logarithmic (log) phase (OD at 0.3–0.4). After verifying the base sequence, the plasmid was washed five times with 10% glycerol and purified. Subsequently, MAP (K10) competent cells were electroporated, which were subsequently placed in antibiotic-free 7H9 broth for one day and subsequently plated on kanamycin-7H10 agar. After incubating at 37°C for 3–4 weeks, the colonies were transferred to kanamycin-7H9 broth and cultured again. Finally, positive clones were confirmed by PCR and electrophoresis using forward and reverse primers within the CRISPRi vector backbone. Additionally, the sequences of the primers used for the construction of the CRISPRi plasmids are provided in Table 1.

**TABLE 1:** Primers used in the study^*a*^

	Primer name	Sequence (5'- 3')
CRISPRi cloning		
*mdh* (Malate dehydrogenase)	MAP2541c 145F	GGGA* GCCGATCTCGACGCCGGACAGCAG
	MAP2541c 145R	AAAC** CTGCTGTCCGGCGTCGAGATCGGC
	MAP2541c CtrlF	**GGGA** GGTTGTGGTCCAGGCGGGTCAGC
	MAP2541c CtrlR	AAAC GCTGACCCGCCTGGACCACAACC
MAP1981c (Putative nucleic acid-binding protein)	MAP1981c 216F	**GGGA** GACCGGACCCCTTGACCCGCA
	MAP1981c 216R	AAAC TGCGGGTCAAGGGGTCCGGTC
	MAP1981c CtrlF	**GGGA** GCACCACGTCGTCCTCGGCGGCC
	MAP1981c CtrlR	AAAC GGCCGCCGAGGACGACGTGGTGC
*pknG* (Protein kinase G)	MAP3893c 216F	**GGGA** GCCCCACCGGTTTGCCGCAGTTCCA
	MAP3893c 216R	AAAC TGGAACTGCGGCAAACCGGTGGGGC
	MAP3893c CtrlF	**GGGA** GGTTCCGGCTGCTCGCTCTT
	MAP3893c CtrlR	AAAC AAGAGCGAGCAGCCGGAACC
*icl* (Isocitrate lyase)	MAP3961 216F	**GGGA** CTCGAGGCCGTTACGGACCCG
	MAP3961 216R	AAAC CGGGTCCGTAACGGCCTCGAG
	MAP3961 CtrlF	**GGGA** TGTTGCCGGTCAGGGCGCCC
	MAP3961 CtrlR	AAAC GGGCGCCCTGACCGGCAACA
CRISPRi vector backbone		
PLJR965	CRISPRi-confirm F	CAGTCGAAAGACTGGGCCTT
	CRISPRi-confirm R	AGCTTCCTGTGAAGAGCCAT
qRT-PCR		
*mdh* (Malate dehydrogenase)	MAP2541c_qRT_F	GATGGAACTCGACGACTGCG
	MAP2541c_qRT_R	TCGAAGATCTTGTTCGGGTC
MAP1981c (Putative nucleic acid-binding protein)	MAP1981c_qRT_F	TGGAAATCGACCGCGGGGAG
	MAP1981c_qRT_R	TCACTGACCGGACCCCTTGA
*pknG* (Protein kinase G)	MAP3893c_qRT_F	GCGAGGCGCTGATGACCAAC
	MAP3893c_qRT_R	CCCTTGCTCTTCTTGGTGGA
*icl* (Isocitrate lyase)	MAP3961_qRT_F	CGCACCAAGGAGGGCTTCTA
	MAP3961_qRT_R	GTCTCCATCCAGATCAGGTC

^*a*^ * indicates sgRNA promoter sequences (bold), and ** indicates dCas9 handle sequences (underlined).

### Quantification of gene expression levels in MAP mutants

MAP mutants were grown in kanamycin-7H9 broth at 37°C. Each mutant was separated into two groups: one group with induction by adding ATc and the other group without induction as a control. After the infection of THP-1 macrophages with MAP mutants, the gene expression level of each mutant within the THP-1 macrophage population was measured on Day 3 after the addition of ATc. Total bacterial RNA was extracted using an RNeasy Mini Kit (Qiagen, Hilden, Germany), following the manufacturer’s instructions with modifications, which was a mechanical disruption of MAP cell wall with 0.1-mm zirconia/silica beads. The quality of the extracted RNA was assessed using A_260/230_ and A_260/280_ ratios with an ND–1000 Spectrophotometer (NanoDrop, Waltham, MA, USA). Subsequently, cDNA was synthesized using a high-capacity cDNA reverse transcription kit (Applied Biosystems, Foster City, CA, USA), and amplified using gene-specific primers (Table 1). RT‒qPCR was carried out via real-time qPCR with 2× Master Mix (SYBR Green, Elpisbiotech, Daejeon, Republic of Korea), and the genes were amplified with a Rotor-Gene Q real-time PCR cycler (Qiagen, Hilden, Germany). The gene expression levels were determined through the 2^−ΔΔCt^ method with *sigA* as a reference gene. The fold change was determined on the basis of the relative gene expression level compared with that of the control.

### Viability of MAP mutants

Viability of MAP mutants was measured by colony-forming unit (CFU) of the bacteria on kanamycin-7H10 agar. To optimize the biomass of the MAP mutant to be used in this study, the standard for the growth section of the MAP mutant was set based on the growth curve, and the 0.5 at OD_600_, which is the section where the bacterial number increases, was set as the log phase. ATc was added when the growth curve reached the log phase, and each culture was measured at 3 days intervals up to 12 days using CFU counts.

### Cell culture and infection

The human monocytic cell line THP-1 was cultured in RPMI 1640 supplemented with 10% heat-inactivated FBS (Gibco) and 1% penicillin/streptomycin at 37°C with 5% CO_2_. THP-1 cells were differentiated into macrophages by stimulation with 50 ng/mL phorbol 12-myristate 13-acetate (PMA) (Sigma Co., St. Louis, MO, USA). After incubation for 72 h, the differentiated cells were washed twice with FBS-free RPMI 1640 medium and incubated with 5% FBS-RPMI 1640 medium without antibiotics for 24 h before infection. To perform the infection, the bacterial clumps were dispersed by passing through a 22G needle 10 times after vortexing of the broth culture. Dead or clumped bacteria were removed by standing the culture for 5 min after the treatment. A total of 5 × 10^5^ THP-1 cells were inoculated with MAP mutants at a multiplicity of infection (MOI) of 10:1 and incubated for 3 h. After incubation for 3 h, the cells were washed twice with 1× DPBS to remove noninternalized bacteria.

RPMI medium supplemented with 50 µg/mL kanamycin or ATc at various concentrations was added to the infected cells, which were subsequently incubated at 37°C with 5% CO_2_. After 1–3 days, the infected cells were lysed with 0.1% Triton X-100 (Sigma‒Aldrich, MO, USA) for 15 min at room temperature to determine the number of CFU/mL on Middlebrook 7H10 agar supplemented with 50 µg/mL kanamycin. To determine THP-1 macrophage survival under MAP mutant infection, 72 h post-infection (h.p.i.), the THP-1 cells were washed with 1× DPBS, and a WST-8 assay was performed on the infected cells as described below. THP-1 macrophage survival was determined by comparison with that of no ATc-treated infected macrophages (containing all the supplements used, excluding ATc).

### THP-1 macrophage WST-8 assay

To exclude adverse effects of ATc on THP-1 cells, assays were performed using a WST-8 cell viability assay kit (DyneBio, Seongnam, Republic of Korea). THP-1 cells were seeded in 96-well plates at 1 × 10^5^ cells/well in 10% inactivated FBS-RPMI 1640 medium supplemented with 50 ng/mL PMA and incubated at 37°C with 5% CO_2_ to allow for the cells to adhere and differentiate for 72 h. Stabilization was subsequently performed with 5% FBS-RPMI 1640 medium for 24 h. Following stabilization, the medium in the plate was discarded, and 10-µL dilutions of ATc prepared in 5% FBS-RPMI (1–100 µg/mL) were added to the cells, which were incubated for an additional 24 h. Following the 24-h incubation, 5% RPMI was discarded, and cells were washed twice in pre-warmed sterile PBS gently. After treatment, 10 µL of WST-8 solution was added to each well, and the THP-1 cells were incubated for an additional 4 h at 37°C in a CO_2_ incubator. After the reaction, gently shake for 1 min before measuring the absorbance. The absorbance was measured with a VersaMax microplate reader (Molecular Devices Corporation, CA, USA) at 450 nm. The percentage of cell viability was determined by comparing the values of treated and untreated cells.

### Transmission electron microscopy (TEM)

For TEM analysis, infected cells were washed with 0.05 M sodium cacodylate buffer (pH 7.4). After several washes, the cells were fixed with 1% (w/v) osmium tetroxide for 1 h at room temperature. The fixed cells were washed with distilled water and stained with 0.5% (w/v) uranyl acetate (in water) at 4°C overnight. The stained cells were washed several times with distilled water and dehydrated in 30%, 50%, 70%, 80%, 90%, and 100% ethanol for 20 min prior to being embedded in Spurr’s resin. The dehydrated samples were immersed in a mixture of 100% ethanol and Spurr’s resin (1:1) for 2 h, 100% ethanol and Spurr’s resin (1:2) for 2 h, Spurr’s resin at 4 ℃ for 24 h, and Spurr’s resin at 25 ℃ for 3 h. Each sample was polymerized at 70 ℃ for 24 h and sectioned using an ultramicrotome (EM UC7; Leica, Wetzlar, Germany). The thinly sliced sections were subjected to TEM (energy-filtered [EF]-TEM) (JEM-1010; JEOL, Japan) at an acceleration voltage of 80 kV (59).

### Statistical analysis

The data from all experiments were statistically analyzed. The statistical significance of the differences was analyzed by repeated-measures ANOVA using GraphPad Prism software version 9.4.0 (GraphPad Software, San Diego, CA, USA; https://www.graphpad.com). Additionally, the data are presented as the means ± standard deviations (SDs). The statistical significance of differences was determined via ANOVA with Tukey’s multiple comparisons test, and a *P*-value less than 0.05 was considered to indicate statistical significance. For calculations containing ODs and CFUs, logarithmic transformations were applied. All the experiments were performed in triplicate.

## SUPPLEMENTAL MATERIAL

Table S1 jb.00244-25-s0001.docx qRT-PCR-based fold change in gene expression of MAP knockdown mutants in the presence and absence of ATc.

ASM does not own the copyrights to Supplemental Material that may be linked to, or accessed through, an article. The authors have granted ASM a non-exclusive, world-wide license to publish the Supplemental Material files. Please contact the corresponding author directly for reuse.
