# Supplementary material for: Functional analysis of the intracellular survival of Mycobacterium avium subsp. paratuberculosis in THP-1 cells using CRISPR interference
Source: J Bacteriol. 2025 Sep 12;207(10):e00244-25. doi: 10.1128/jb.00244-25 (PMC12548404; doi:10.1128/jb.00244-25)
Supplement: Table S1 — qRT-PCR-based fold change in gene expression of MAP knockdown mutants in the presence and absence of ATc. [file jb.00244-25-s0001.docx]

**Table S1. qRT-PCR–based fold change in gene expression of MAP knockdown mutants in the presence and absence of ATc.**

| **Gene** | **Fold Change (-ATc)** | **Fold Change (+ATc)** | **n** | **Description** |
| --- | --- | --- | --- | --- |
| *mdh* | 1.06 ± 0.09 | 0.27 ± 0.02 | 3 | Malate dehydrogenase |
| *pknG* | 1.08 ± 0.12 | 0.28 ± 0.11 | 3 | Protein kinase G |
| *MAP1981c* | 1.08 ± 0.12 | 0.28 ± 0.03 | 3 | Putative Nucleic Acid-Binding Protein |
| *icl* | 1.04 ± 0.07 | 0.30 ± 0.03 | 3 | Isocitrate lyase |
